# Defect Chemistry and Li-ion Diffusion in Li_2_RuO_3_

**DOI:** 10.1038/s41598-018-36865-4

**Published:** 2019-01-24

**Authors:** Navaratnarajah Kuganathan, Apostolos Kordatos, Alexander Chroneos

**Affiliations:** 1 Department of Materials, Imperial College London, London, SW7 2AZ United Kingdom; 2 Faculty of Engineering, Environment and Computing, Coventry University, Priory Street, Coventry, CV1 5FB United Kingdom

## Abstract

Layered Li_2_RuO_3_ is an important candidate cathode material in rechargeable lithium ion batteries because of its novel anionic redox process and high reversible capacity. Atomistic scale simulations are used to calculate the intrinsic defect process, favourable dopants and migration energies of lithium ion diffusions together with migration paths in Li_2_RuO_3_. The Li Frenkel is calculated to be the most favourable intrinsic defect type. The cation anti-site defect, in which Li and Ru ions exchange their positions is 1.89 eV/defect suggesting that this defect would be observed at high temperatures. Long range vacancy assisted lithium diffusion paths were calculated and it is confirmed that the lowest overall activation energy (0.73 eV) migration path is along the *ab* plane. Trivalent dopants (Al^3+^, Co^3+^, Sc^3+^, In^3+^, Y^3+^, Gd^3+^ and La^3+^) were considered to create additional Li in Li_2_RuO_3_. Here we show that Al^3+^ or Co^3+^ are the ideal dopants and this is in agreement with the experimental studies reported on Co^3+^ doping in Li_2_RuO_3_.

## Introduction

High energy storage systems needed for the development of electronic vehicles and consumer electronics require high-capacity lithium ion battery cathode materials^1–5^. The development of such materials has many challenges such as materials being safe, with low cost and high abundance. A variety of new cathode materials^6–22^ have been studied both experimentally and theoretically though a few of them have been identified as promising. There is a continuous research activity by considering those challenges to find new cathode materials to improve the power density in Li ion batteries.

“Layered” Li_2_RuO_3_ has attracted attention because of its novel anionic redox process^23^. Reversible oxygen redox process is a key feature in Li_2_RuO_3_ and enhances the capacity of this material^23^. Experimental studies^24–26^ demonstrate that extraction of both lithium is possible but one of them can be repeatedly cycleable. Moore *et al*.^24^ studied the electrochemical properties of Li_2_RuO_3_ and concluded that there are two working plateaus in the first charging process providing a reversible capacity of approximately 270 mAhg^−1^. A novel hybrid Na^+^/Li^+^ battery has been recently made using Li_2_RuO_3_ as a cathode material because of its unique structure accommodating both Li^+^ and Na^+^ ions^27^. Li_2_RuO_3_ was suggested as an additive to provide high energy lithium-ion capacitors due to its high reversible characteristics for Li^+^ ion intercalation/de-intercalation and structural stability^28^. Recently, Arunkumar *et al*.^29^ synthesized over-lithiated Li_2+x_Ru_1−x_CoO_3_ cathode by aliovalent Co doping on Ru site in Li_2_RuO_3_ and concluded that there is an enhancement in the electrochemical lithium reversibility and Li^+^ extraction compared to those associated in the pristine Li_2_RuO_3_.

Electrochemical behaviour of an electrode material by studying its defect properties is important to assess its applicabilty in batteries. Computational modelling can provide useful information of the key issues related to defect processes including cation mixing and doping strategies to increase the Li concentration in this material. In a vast range of oxides including these Li-based systems classical pair potentials do capture the trends and energetics of the defect processes in excellent agreement with experiment^30–32^. For example, the lithium ion migration path calculated in LiFePO_4_ using classical pair potentials^33^ was exactly observed later in the neutron diffraction experiment^34^. Here, we extend our recent simulation studies of the Li_5_FeO_4_^18^, Li_2_CuO_2_^22^, Li_9_V_3_(P_2_O_7_)_3_(PO_4_)_2_^35^ and Li_2_SnO_3_^36^ electrode materials where we investigated the defects, lithium ion diffusion and dopants. In this study, we have systematically studied the relative energetics of the formation of intrinsic defects, the solution of trivalent dopants (Al^3+^, Co^3+^, Sc^3+^, In^3+^, Y^3+^, Gd^3+^ and La^3+^), and the possible lithium ion migration pathways in Li_2_RuO_3_.

## Results and Discussion

### Li_2_RuO_3_ structure

**Figure 1:**
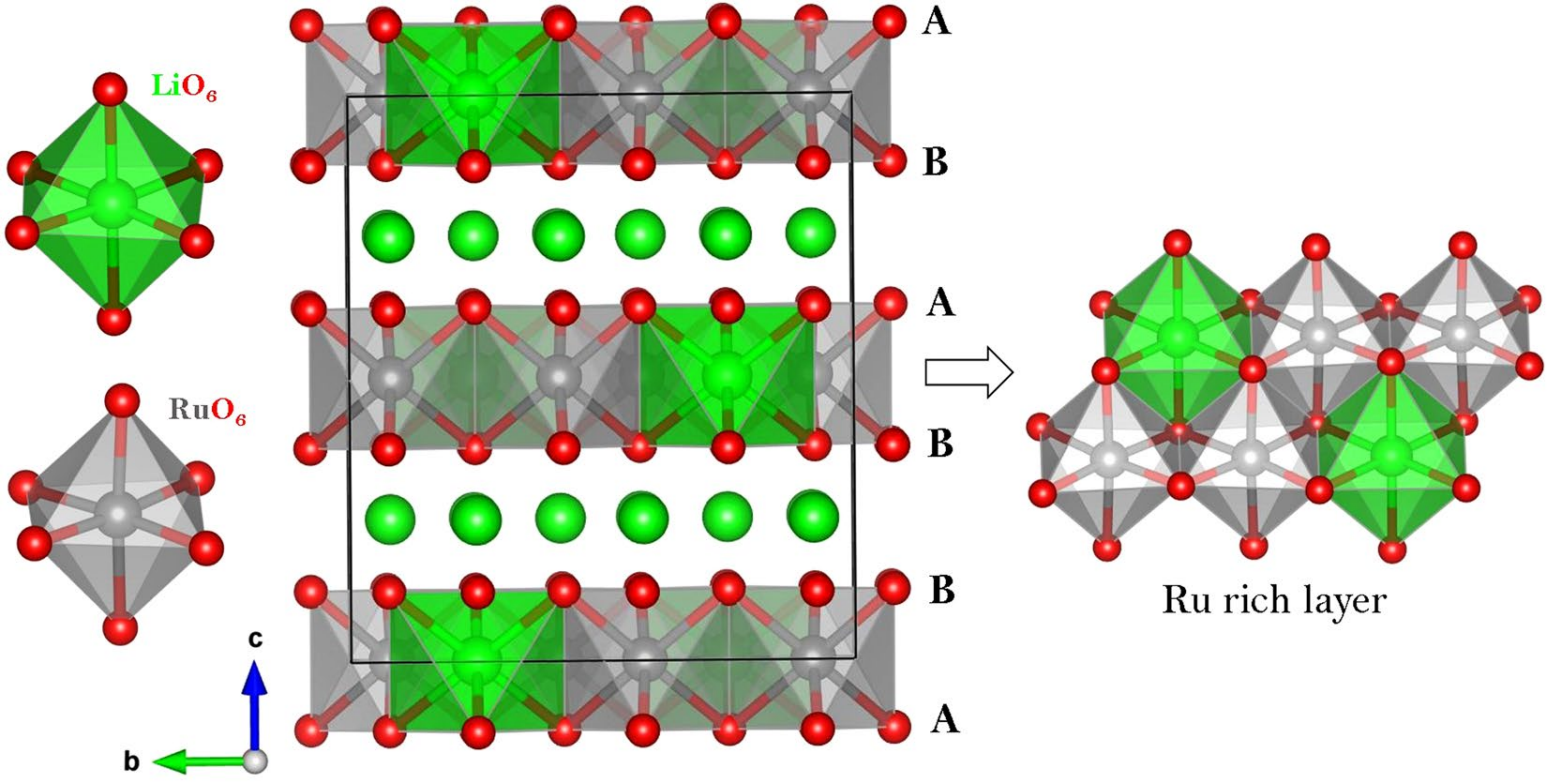
Crystal structure of Li_2_RuO_3_ (space group C2/c).

Li_2_RuO_3_ is a layered structure and has a monoclinic symmetry with space group C2/c. Its experimental lattice parameters (a = 4.9230 Å, b = 8.7746 Å, c = 9.8776 Å, α = 90°, β = 100.073° and γ = 90°) was reported by Kobayashi *et al*.^26^. Figure 1 exhibits this structure, the coordination environments of Ru and Li (both forming octahedrons with six O atoms) and layers in the *ab* plane with an A-B stacking sequence (P2 type) as classified by Delmas *et al*.^37^. First, experimentally observed monoclinic crystal structure was reproduced to assess the quality of the classical pair potentials (potentials parameters are reported in Table S1 in the supplementary information) used in this study. There is a good agreement between the calculated equilibrium lattice constants (tabulated in Table 1) and the experimental values.

**Table 1:** Calculated and Experimental Structural Parameters for monoclinic (C2/c) Li_2_RuO_3_.

Parameter	Calc	Expt^26^	|∆|(%)
a (Å)	5.0622	4.9230	2.83
b (Å)	8.7521	8.7746	0.26
c (Å)	9.9749	9.8776	0.99
α (°)	90.0	90.0	0.00
β (°)	99.593	100.073	0.48
γ (°)	90.0	90.0	0.00

### Intrinsic defect processes

As defect properties of an electrode material is important to understand its electrochemical behavior, we calculated a series of isolated point defect (vacancy, anti-site and interstitial) energies. Frenkel, Schottky and anti-site defect formation energies were then calculated by combining the isolated point defects. Here we use Kröger-Vink notation^38^ to represent the reactions involving these defects.

1 $${\rm{Li}}\,{\rm{Frenkel}}:{{\rm{Li}}}_{{\rm{Li}}}^{{\rm{X}}}\to {V}_{{\rm{Li}}}^{{\prime} }+{{\rm{Li}}}_{{\rm{i}}}^{\bullet }$$

2 $${\rm{O}}\,{\rm{Frenkel}}:{{\rm{O}}}_{{\rm{O}}}^{{\rm{X}}}\to {V}_{{\rm{O}}}^{\bullet \bullet }+{{\rm{O}}}_{{\rm{i}}}^{{\prime\prime} }$$

3 $${\rm{Sn}}\,{\rm{Frenkel}}:{V}_{{\rm{Ru}}}^{{\rm{X}}}\to {V}_{{\rm{Ru}}}^{{\prime\prime} \,{\prime\prime} }+{{\rm{V}}}_{{\rm{i}}}^{\bullet \bullet \bullet \bullet }$$

4 $${\rm{Schottky}}:2\,{{\rm{Li}}}_{\mathrm{Li}\,}^{{\rm{X}}}+{{\rm{Ru}}}_{{\rm{Ru}}}^{{\rm{X}}\,}+3{{\rm{O}}}_{{\rm{O}}}^{{\rm{X}}}\to 2{V}_{{\rm{Li}}}^{\text{'}}+{V}_{{\rm{Ru}}}^{{\prime\prime} \,{\prime\prime} }+3{V}_{{\rm{O}}}^{\bullet \bullet }+{{\rm{Li}}}_{2}{{\rm{RuO}}}_{3}$$

5 $${{\rm{Li}}}_{2}{\rm{O}}\,{\rm{Schottky}}:2\,{{\rm{Li}}}_{{\rm{Li}}}^{{\rm{X}}}+{{\rm{O}}}_{{\rm{O}}}^{{\rm{X}}\,}\to 2{V}_{{\rm{Li}}}^{\text{'}}+{V}_{{\rm{O}}}^{\bullet \bullet }+{{\rm{Li}}}_{2}{\rm{O}}$$

6 $${\rm{Li}}/{\rm{Ru}}\,{\rm{antisite}}\,({\rm{isolated}}):{{\rm{Li}}}_{{\rm{Li}}}^{{\rm{X}}}+{V}_{{\rm{Ru}}}^{{\rm{X}}\,}\to {{\rm{Li}}}_{{\rm{Ru}}}^{{\prime} {\prime} {\prime} }+{{\rm{Ru}}}_{{\rm{Li}}}^{\bullet \bullet \bullet }$$

7 $${\rm{Li}}/{\rm{Ru}}\,{\rm{antisite}}\,({\rm{cluster}}):{{\rm{Li}}}_{{\rm{Li}}}^{{\rm{X}}}+{{\rm{Ru}}}_{{\rm{Ru}}}^{{\rm{X}}}\to \{{{\rm{Li}}}_{{\rm{Ru}}}^{{\prime} {\prime} {\prime} }:{{\rm{Ru}}}_{{\rm{Li}}}^{\bullet \bullet \bullet }\}{\rm{x}}$$

**Figure 2:**
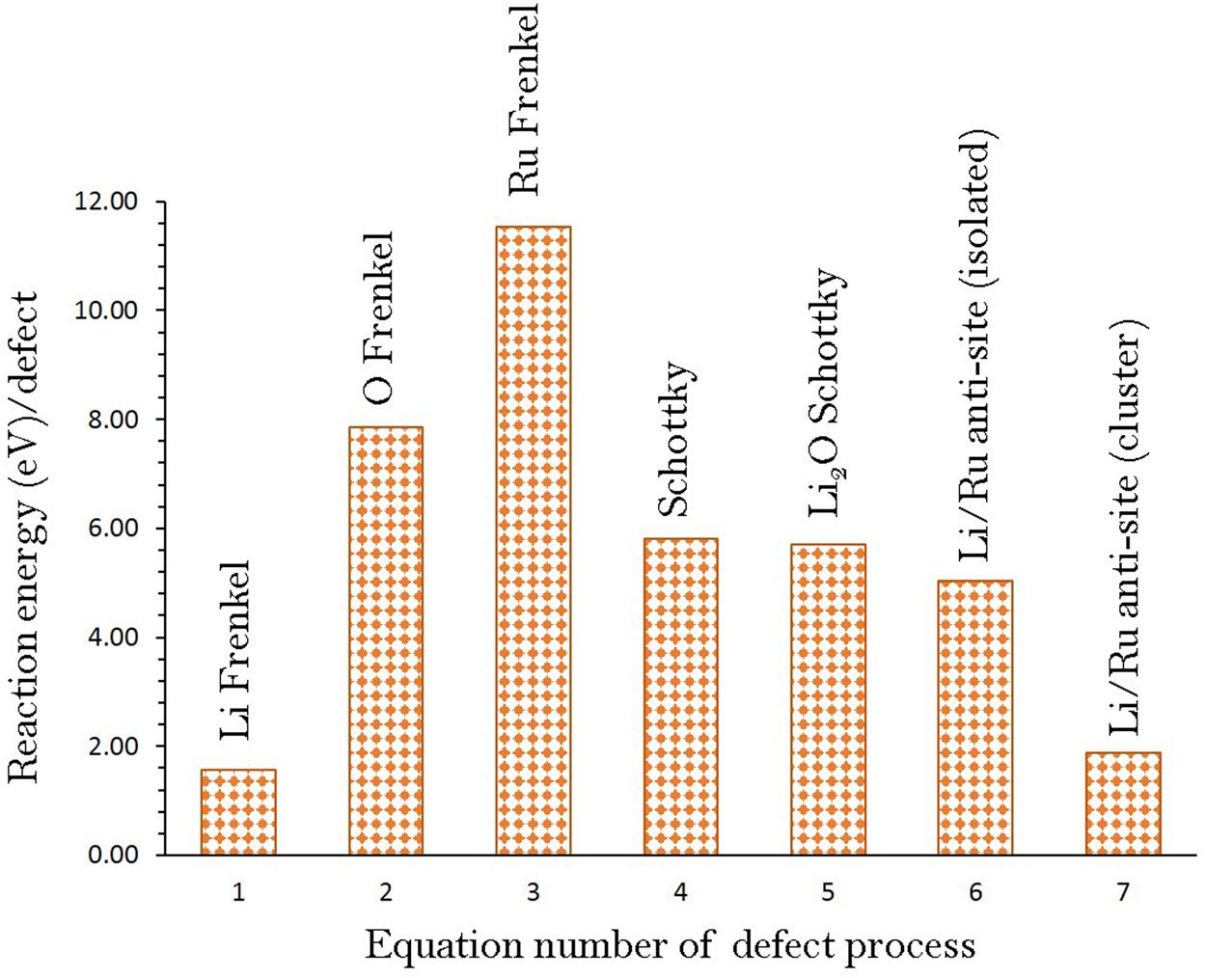
Energetics of intrinsic defect process in monoclinic Li_2_RuO_3_.

Figure 2 reports the reaction energies for these intrinsic defect processes. The most favorable intrinsic disorder is found to be the Li Frenkel. Formation of other Frenkel and Schottky defects is thermodynamically unfavorable. The second most favorable defect process is the anti-site suggesting that there will be a small percentage of Li on Ru sites ${(\mathrm{Li}}_{{\rm{Ru}}}^{{\prime} {\prime} {\prime} })$ and Ru on Li sites ${(\mathrm{Ru}}_{{\rm{Li}}}^{\bullet \bullet \bullet })$. However, this defect would not be observed at operating temperatures. This defect has been observed experimentally and theoretically in a variety of Li ion battery materials^6,9,39–42^. The formation of other Frenkels (Ru and O) and Schottky defects is found to be unfavorable.

### Lithium ion-diffusion

**Figure 3:**
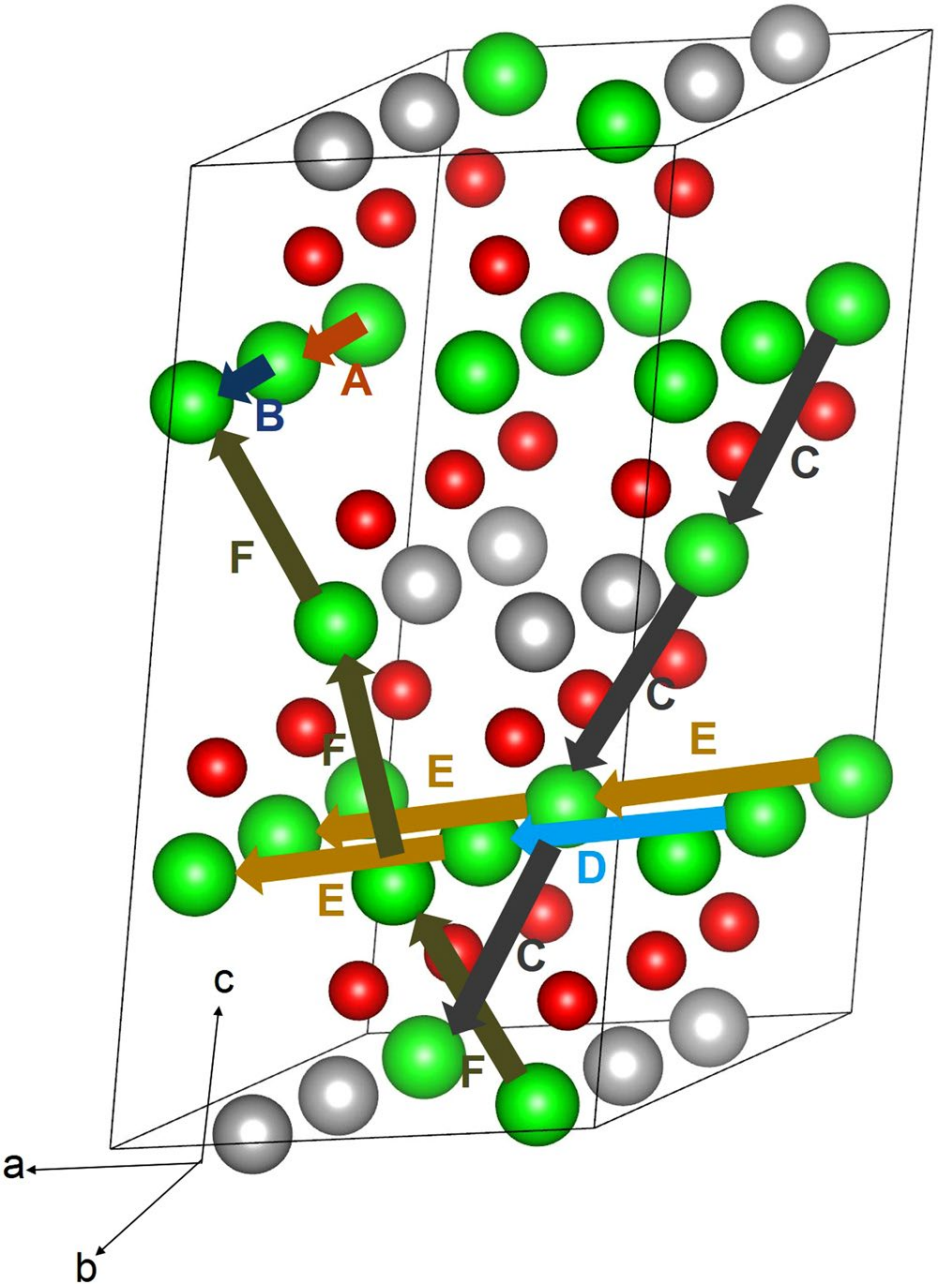
Possible long range lithium vacancy migration paths considered. Green, grey and red colors correspond to Li, Ru, and O atoms respectively.

**Figure 4:**
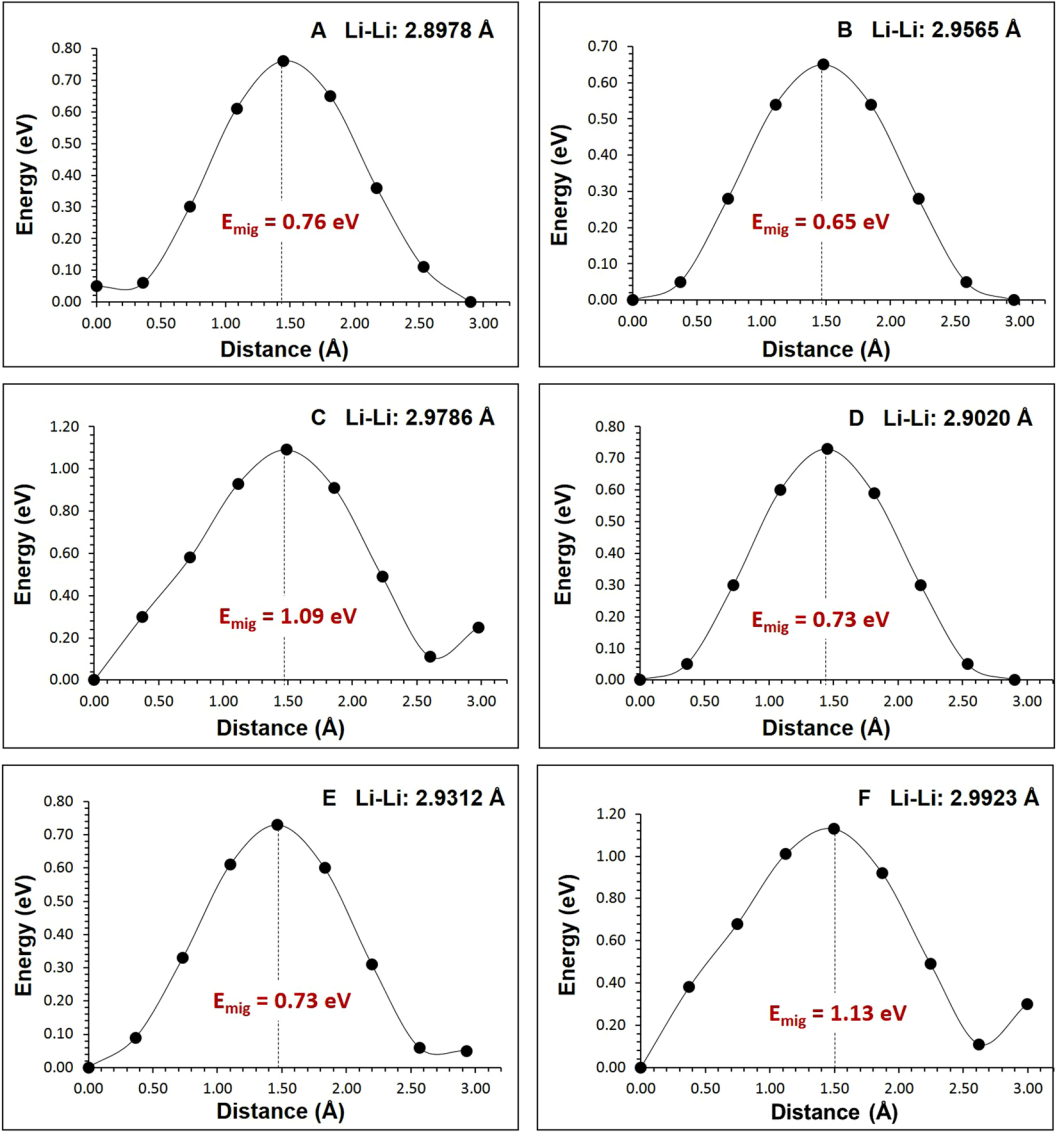
Six different energy profiles [as shown in Fig. 3] of Li vacancy hopping between two adjacent Li sites in Li_2_RuO_3_.

Diffusion of lithium ion diffusion with lower activation energy is a key requirement for a promising cathode materials in lithium ion batteries. Static atomistic simulation allows us to examine various possible Li ion migration paths that are in general diffucly by experimetanl studies alone. For the Li vacancy migration, we have calculated six different local Li hops (refer to Fig. 3). Table 2 reports the migration energies together with the Li-Li separation, whereas energy profile diagrams are shown in Fig. 4. We have constructed long range paths connecting local Li hops with lower overall activation energy. We have identified five long range paths (refer to Fig. 3). The first long range path exhibits a linear path (A → B → A → B) along *b* axis consisting of a local Li hop with lower activation energy of migration of 0.65 eV (local hop B) but with overall activation energy of 0.76 eV (refer to Table 3). The second path exhibits a zig-zag pattern (C → C → C → C) with an activation energy of 1.09 eV. Both the third and fourth migration paths [D → E → D → E and E → E → E→E] exhibit a linear path along *ab* plane with the lowest activation energy of 0.73 eV. The fifth migration path (F → F → F → F) is constructed along *ac* plane and its activation energy is calculated to be 1.13 eV. Here we define the highest potential energy along the migration path as the activation energy.

**Table 2:** Calculated Li-Li separations and activation energies for the lithium ion migration between two adjacent Li sites refer to Fig. 3.

Migration path	Li-Li separation (Å)	Activation energy (eV)
A	2.8978	0.76
B	2.9565	0.65
C	2.9786	1.09
D	2.9020	0.73
E	2.9312	0.73
F	2.9923	1.13

**Table 3:** Possible long range Li ion diffusion paths and their corresponding overall activation energies.

Long range path	Overall activation energy (eV)
A → B → A → B	0.76
C → C → C → C	1.09
D → E → D → E	0.73
E → E → E → E	0.73
F → F → F → F	1.13

### Trivalent doping

The capacity of a cathode material can be increased by incorporating additional lithium into the as-prepared material. An efficient way to increase the amount of lithium is by doping trivalent cations on Ru site through creating Li interstitials. The experimental study^29^ on Co^3+^ doping on Ru site reveals that the resultant Li_2.1_Ru_0_._9_Co_0.1_O_3_ exhibits significant reversible Li^+^ extraction compared to undoped Li_2_RuO_3_. Similar approach has been previously demonstrated computationally in Li_2_MnSiO_4_ cathode material^12^. Here we considered the solution of ${R}_{2}{O}_{3}$ (*R* = Al, Co, Sc, In, Y, Gd and La) via the following process (in Kröger-Vink notation):

8 $${{\rm{R}}}_{2}{{\rm{O}}}_{3}+2{{\rm{Ru}}}_{{\rm{Ru}}}^{{\rm{X}}}+{{\rm{Li}}}_{2}{\rm{O}}\to 2{{\rm{R}}}_{{\rm{Ru}}}^{\text{'}}+2{{\rm{Li}}}_{{\rm{i}}}^{\bullet }+2{{\rm{RuO}}}_{2}$$

**Figure 5:**
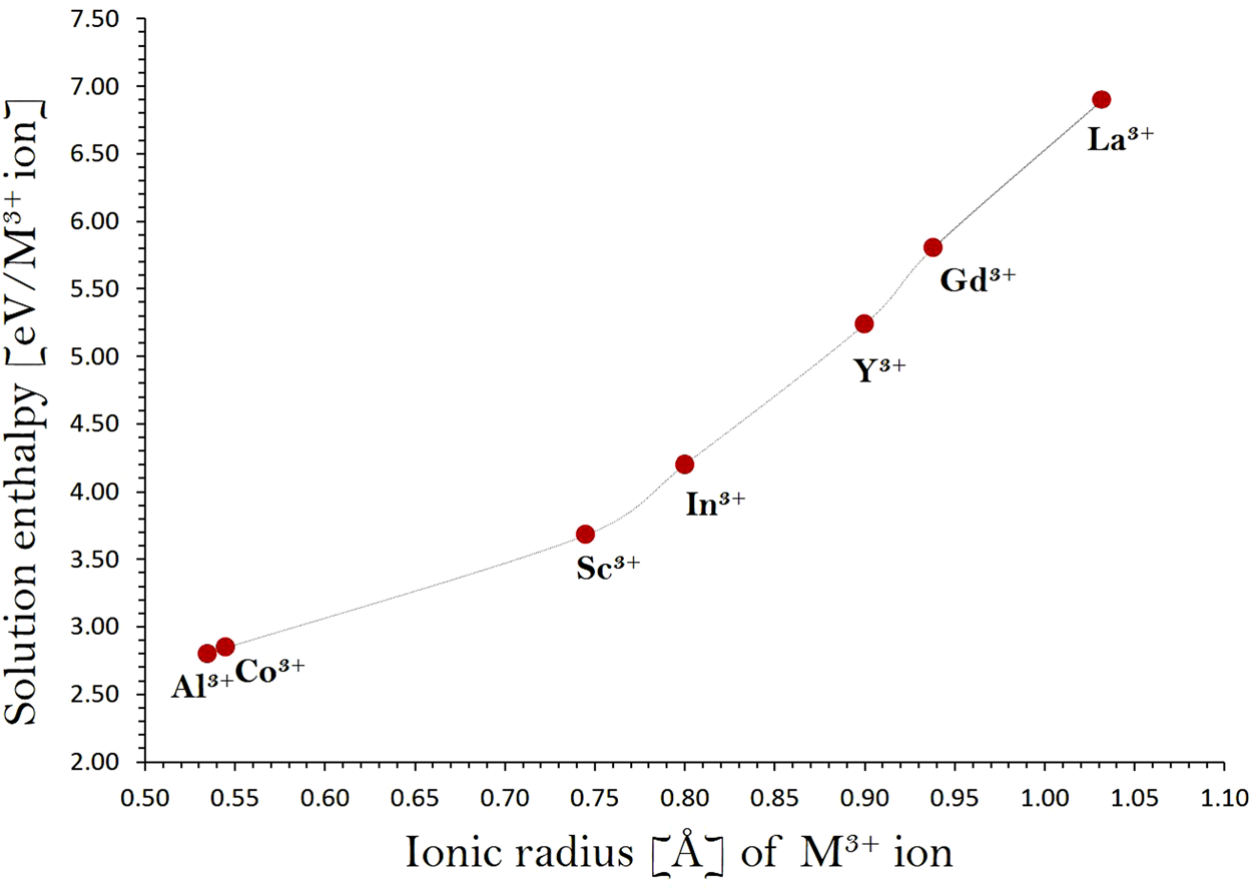
Enthalpy of solution of ${{\rm{R}}}_{2}{{\rm{O}}}_{3}$ (*R* = Al, Co, Sc, In, Y, Gd and La) with respect to the R^3+^ ionic radius in Li_2_RuO_3_.

The solution energies of ${R}_{2}{O}_{3}\,\,$are reported in Fig. 5. The most favorable dopant is found to be Al^3+^. The solution energy for Co_2_O_3_ is higher in energy by only 0.05 eV suggesting that Co^3+^ is also a candidate dopant to increase the Li concentration in Li_2_RuO_3_. Our calculation confirms the experimental investigation^29^ reported for Co^3+^ doping and suggests that Al^3+^ is also a promising dopant for the formation extra lithium into Li_2_RuO_3_. The exact composition of the Al incorporated structure should be investigated experimentally. The calculated solution energies are positive for Al_2_O_3_ and Co_2_O_3_ suggesting that doping can be carried out only at high temperatures. This is further supported by the higher temperature (~1100 °C for 12 hours) used for the synthesis of Co-doped Li_2_RuO_3_.

**Figure 6:**
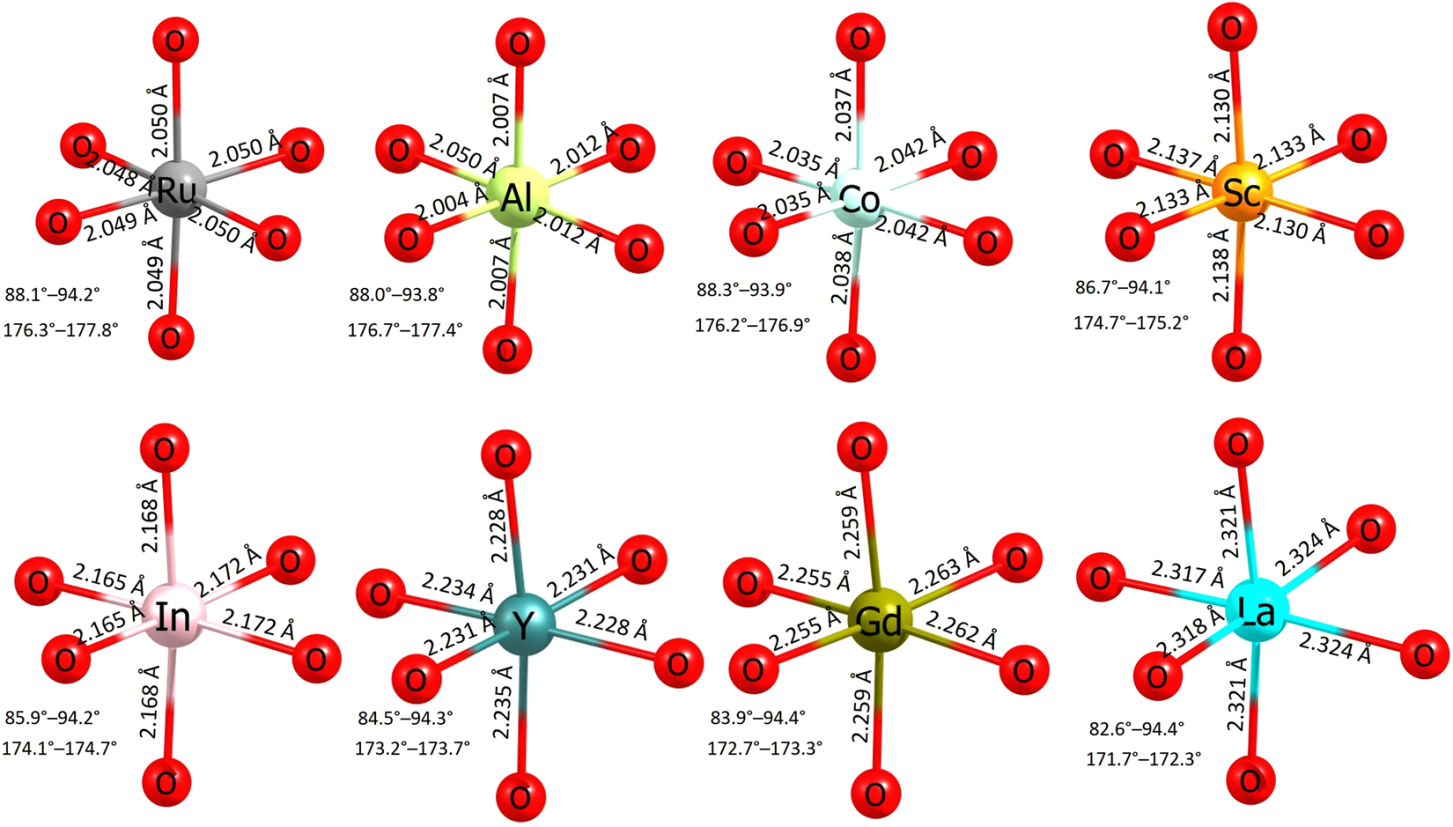
Octahedral RuO_6_ unit in the relaxed structure of undoped Li_2_RuO_3_ and the coordination formed by the dopants on the Ru site with neighbor oxygen.

A detail information regarding the bond lengths and bond angles of dopant and Ru in the relaxed structure of undoped Li_2_RuO_3_ with adjacent oxygens is reported in Fig. 6. The ionic radius of Ru^4+^ in octahedral environment is 0.62 Å, larger by 0.08 Å than that of Al^3+^. In the AlO_6_ unit, all six Al-O bonds are slightly shorter compared to the Ru-O bonds present in the undoped Li_2_RuO_3_. This is due to its smaller cation size of Al^3+^ which strongly polarises the oxygen ions forming strong ionic bonds with O atoms. The ionic radius of Co^3+^ (0.55 Å) is very close to that of Al^3+^. This is reflected in the bond lenghts and bond angles. From Sc to La, dopant-oxygen bond distances increase and bond angles decrease gradually indicating the structural distortion and reflecting in the solution enthalpies. The LaO_6_ unit exhibits approximately the same La-O bond length, but longer by ~0.30 Å than Ru-O bond length present in RuO_6_. The ionic radius of La^3+^ is larger by 0.28 Å than that of Ru^4+^. This reflects in the extremely high solution enthalpy.

### Density of states

**Figure 7:**
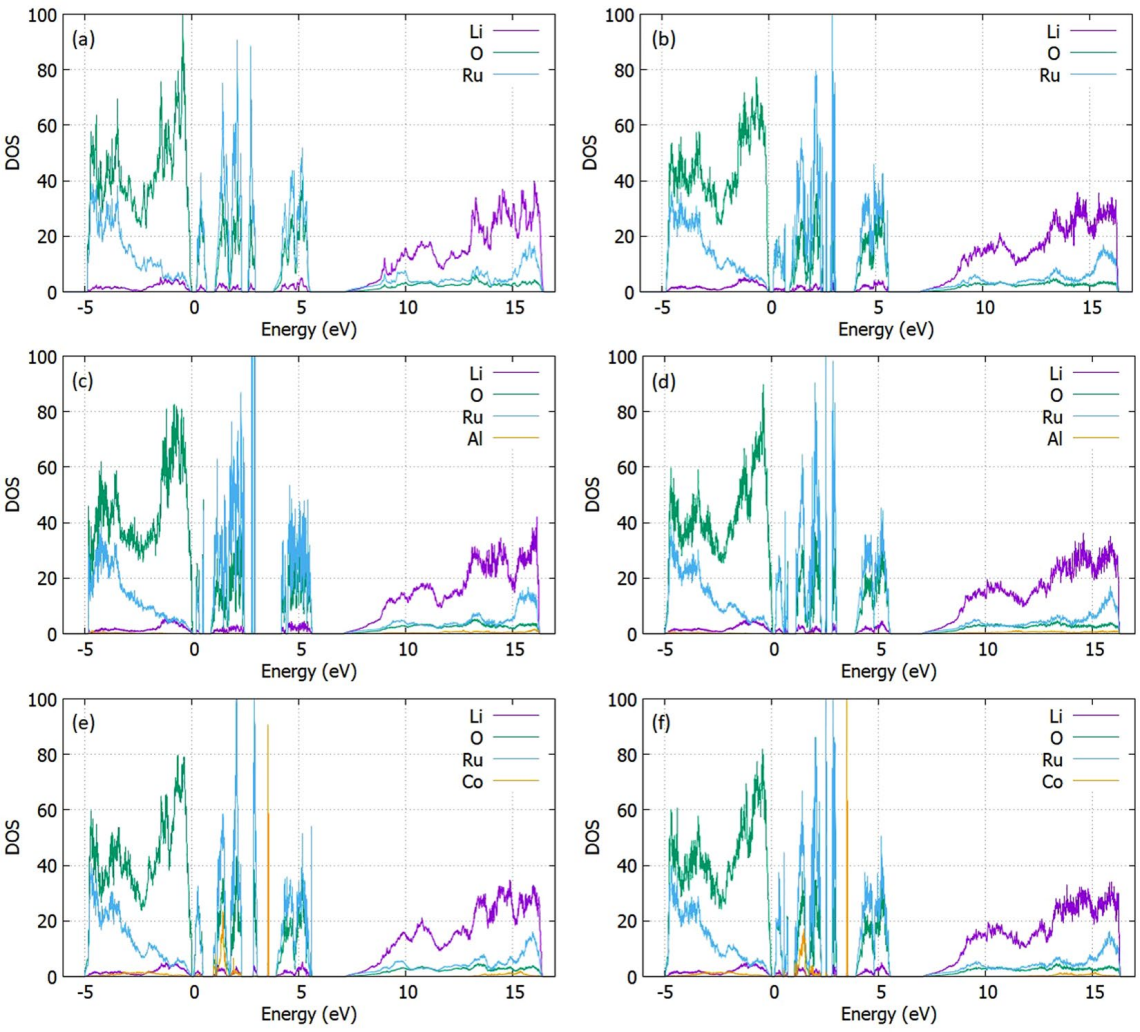
Li_2_RuO_3_ PDOS for (**a**) The non – defective cell (**d**) The Li interstitial (**c**) The Al – doped cell (**d**) The Al – doped cell with one Li interstitial (**e**) The Co – doped cell (**f**) The Co – doped cell with one Li interstitial.

Density Functional Theory (DFT) was used to analyze the electronic structure for trivalent doping processes. In particular, the contribution of every element in the Li_2_RuO_3_ crystal is visualized through the partial Density of States (PDOS). Figure 7 presents the cases for the (a) Non – defective structure (b) The Li interstitial (c) The Al – doped supercell (d) The Al – doped supercell with one Li interstitial. Overall, the material presents the formation of the valence band governed by the strong O^2−^ p-states at the zero-shifted Fermi level (refer to figures reported in supplementary information). Additionally, the conduction band is characterized by the major contribution of Ru *d*-states leading to a narrow band gap of approximately 0.2 eV in agreement with other theoretical studies^43,44^ (refer to Fig. 1 of the SI for the exact contribution of each orbital separately). We point at the presence of in gap states mainly attributed to the Ru^4+^ d-states and O^2−^ p-states. This should be addressed and experimentally investigated as the properties of electronic conduction have to be controlled for future energy applications. The last in-gap contribution is located at 1.66 from the valence band, however this non-uniformity points to an interesting behavior that originates from electronic configuration parameters and must be considered for the oxidation and reduction reactions. The presence of a Li interstitial does not affect the total DOS in a considerable way. Doping Li_2_RuO_3_ with trivalent dopants that substitute the Ru^4+^ site introduces additional contributions in the electronic structure. Regarding the lowest solution enthalpies, we focus on the Al^3+^ and Co^3+^ elements. The dopant is initially introduced as substitutional in a Ru^4+^ site presenting minor distortions in the crystal even combined with a Li^+^ ion in an interstitial site. Al^3+^ doping presents a low contribution at the conduction band with no additional states. However, additional contribution is observed for Co – doping, governed by the Co^3+^ d – states and O^2−^ p-states whereas a weaker contribution due to the Ru^4+^ d-states is also observed (Refer to SI for the orbitals profile). Furthermore, doping with elements of bigger radius introduce intense states in the electronic structure except for In^3+^ (Refer to SI for the extra doping processes considered).

### Summary

Classical pair potential simulations were employed to provide relevant information about favourable intrinsic disorder, Li diffusion paths together with activation energies and possible dopants that can be substituted on Ru site to introduce additional Li in the layered Li_2_RuO_3_. There is a good agreement between the calculated and experimental lattice parameters of Li_2_RuO_3_. The Li Frenkel is the lowest energy and thus the dominant defect energy process. Anti-site disorder is calculated to be 1.89 eV/defect suggesting that a small concentration of cation mixing would be observed at high temperatures. The long range Li ion migration path with lowest activation energy (0.73 eV) is found to be along the *ab* plane. Solution energies of ${R}_{2}{O}_{3}$ (*R* = Al, Co, Sc, In, Y, Gd and La) were considered to create extra lithium in this material and found that Al_2_O_3_ or Co_2_O_3_ would be ideal candidates and this is in agreement with the experimental result reported for Co substitution in Li_2_RuO_3_. This interesting study stimulates further experimental work on Al doping.

## Methods

Intrinsic defect formation energies and Li migration paths were calculated using GULP code^45^. This method is based on the classical pair potentials. Ionic crystal lattice is described using Born model and consists of the long-range attractions and short-range repulsive forces in the form of electron-electron repulsion and van der Waals interactions. Buckingham potentials (refer to Table S1) were used to model the short range interactions. Structural optimizations were performed using the Broyden-Fletcher-Goldfarb-Shanno (BFGS) algorithm^46^. Relaxation around point defects and the diffusing ions were modelled using the Mott-Littleton method^47^. This method has been well explained in our previous publications^18,22^. Vacancy assisted Li ion migration was calculated considering seven interstitial Li ions between local Li hops. Activation energy reported in this study is the local maximum energy along the diffusion path. The present calculation is based on the full ionic charge model within the dilute limit. Therefore, the defect energies will be overestimated, however, the relative energies, and the trends will be consistent.

The electronic structure of Li_2_RuO_3_ is investigated through the plane wave DFT code CASTEP^48,49^. We model the perfect and defective supercells with the plane wave basis set with a cut-off energy of 450 eV using a 2 × 2 × 2 Monkhorst-Pack (MP)^50^ k-point grid within a 96-atomic site supercell. The crystallographic configurations have been initially optimized to the energetically favorable ground state under constant pressure conditions. The exchange and correlation term was modelled using the generalized gradient approximation (GGA) parameterized by Perdew, Burke and Ernzerhof (PBE)^51^ with the use of ultrasoft pseudopotentials^52^. The atomic configurations for the doped/undoped and defective supercells were relaxed to the minimum energy for the electronic structure calculations. For the Density of States (DOS) investigation and visualization, we employ the OPTADOS^53,54^ subcode using a 10 × 10 × 10 k-point grid.

## Supplementary information

Supplementary Information
