# Supplementary material for: Defect Chemistry and Li-ion Diffusion in Li2RuO3
Source: Sci Rep. 2019 Jan 24;9:550. doi: 10.1038/s41598-018-36865-4 (PMC6345755; doi:10.1038/s41598-018-36865-4)
Supplement: Supplementary file 1 — Supplementary Information [file 41598_2018_36865_MOESM1_ESM.docx]

**Supporting Information**

**Defect Chemistry and Li-ion Diffusion in Li_2_RuO_3_**

Navaratnarajah Kuganathan^1^, ApostolosKordatos,^2^ and Alexander Chroneos^1,2^

*^1^Department of Materials, Imperial College London, London, SW7 2AZ, United Kingdom2*

*^3^Faculty of Engineering, Environment and Computing, Coventry University, Priory Street, Coventry CV1 5FB, United Kingdom*

Corresponding authors, e-mails: a) n.kuganathan@imperial.ac.uk

b) [alexander.chroneos@imperial.ac.uk](mailto:alexander.chroneos@imperial.ac.uk)

**Table S1**. Interatomic potential parameters used in the atomistic simulations of Li_2_RuO_3_.

Two-body [Φ*_ij_* (*r_ij_*) = *A_ij_* exp (− *r_ij_* /*ρ_ij_*) − *C_ij_ / r_ij_*^6^]

| Interaction | *A* (eV) | *ρ* (Å) | *C* (eV·Å^6^) | Y (e) | K (eV·Å^-2^) |
| --- | --- | --- | --- | --- | --- |
| Li^+^–O^2−^ | 632.1018 | 0.2906 | 0.00 | 1.000 | 99999 |
| Ru^4+^–O^2−^ | 13733.40 | 0.2259 | 0.49 | 4.000 | 99999 |
| O^2−^–O^2−^ | 12420.50 | 0.2215 | 29.07 | –2.96 | 31.00 |
| Al^3+^ - O^2−^ | 1725.20 | 0.28971 | 0.000 | 3.000 | 99999 |
| Co^3+^ - O^2−^ | 1371.71 | 0.3087 | 0.000 | 3.000 | 99999 |
| Sc^3+^ - O^2−^ | 1575.85 | 0.3211 | 0.000 | 3.000 | 99999 |
| In^3+^ - O^2−^ | 1495.65 | 0.3327 | 4.33 | 3.000 | 99999 |
| Y^3+^ - O^2−^ | 1766.40 | 0.33849 | 19.43 | 3.000 | 99999 |
| Gd^3+^ - O^2−^ | 1885.75 | 0.3399 | 20.34 | 3.000 | 99999 |
| La^3+^ - O^2−^ | 2088.79 | 0.3460 | 23.25 | 3.000 | 99999 |

**Table S2****.** Energetics of intrinsic defect process in Li_2_RuO_3_

| Defect process/equation | Reaction energy/eV | Reaction energy per defect/eV |
| --- | --- | --- |
| Li Frenkel /1 | 3.12 | 1.56 |
| O Frenkel /2 | 15.72 | 7.86 |
| Ru Frenkel /3 | 23.08 | 11.54 |
| Schottky /4 | 34.77 | 5.80 |
| Li_2_O Schottky/5 | 11.40 | 5.70 |
| Li/Ru antisite (isolated) /6 | 10.06 | 5.03 |
| Li/Ru antisite (cluster) /7 | 3.79 | 1.89 |


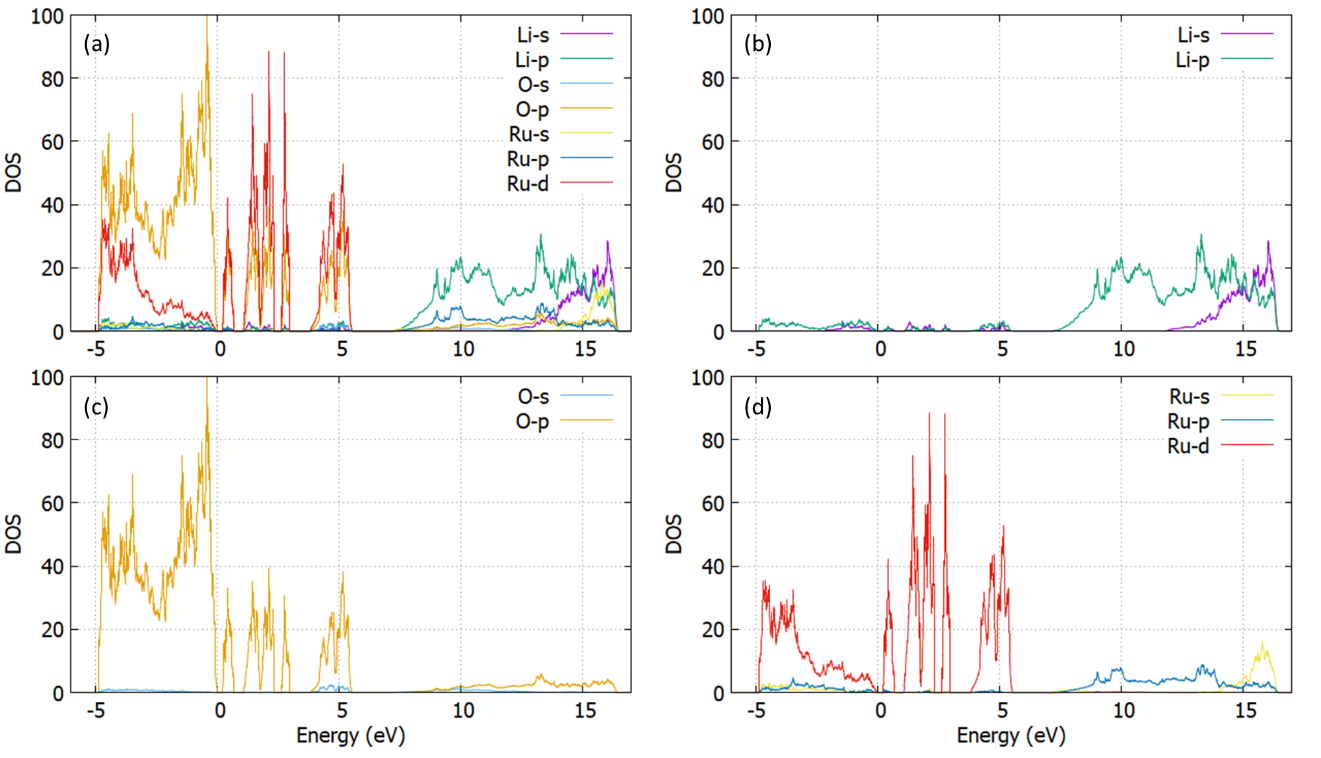


**Figure S1.** Li_2_RuO_3_ PDOS for (a) The total contribution of orbitals in the non – defective cell (b) The contribution of Li^+^ orbitals (c) The contribution of O^2-^ orbitals (d) The contribution of Ru orbitals


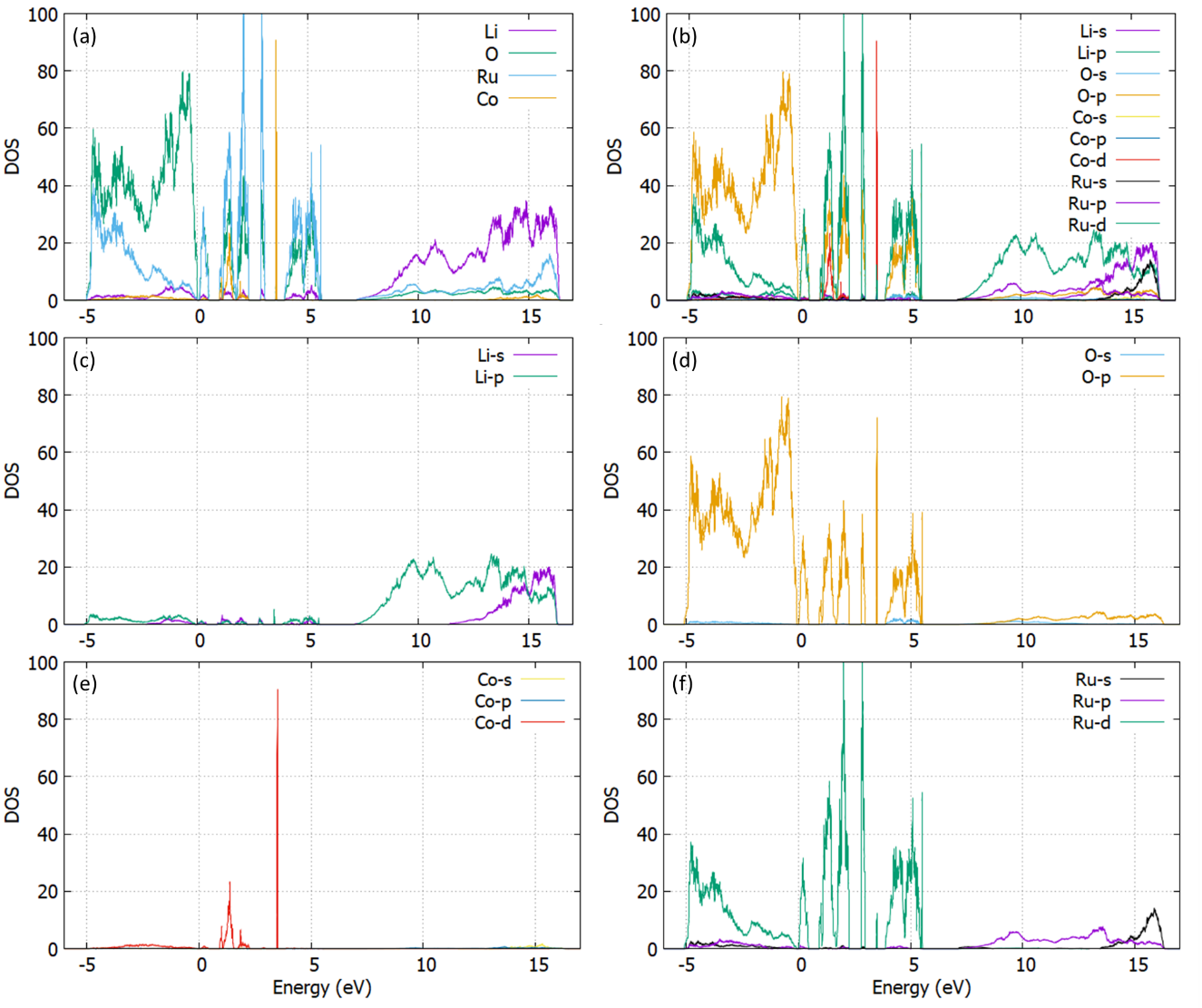


**Figure S2.** The Co – doped Li_2_RuO_3_ PDOS for (a) The contribution of elements in the doped cell (b) The total contribution of orbitals (c) The contribution of Li^+^ orbitals (d) The contribution of O^2-^ orbitals (e) The contribution of Co^3+^ orbitals (f) The contribution of Ru^4+^ orbitals


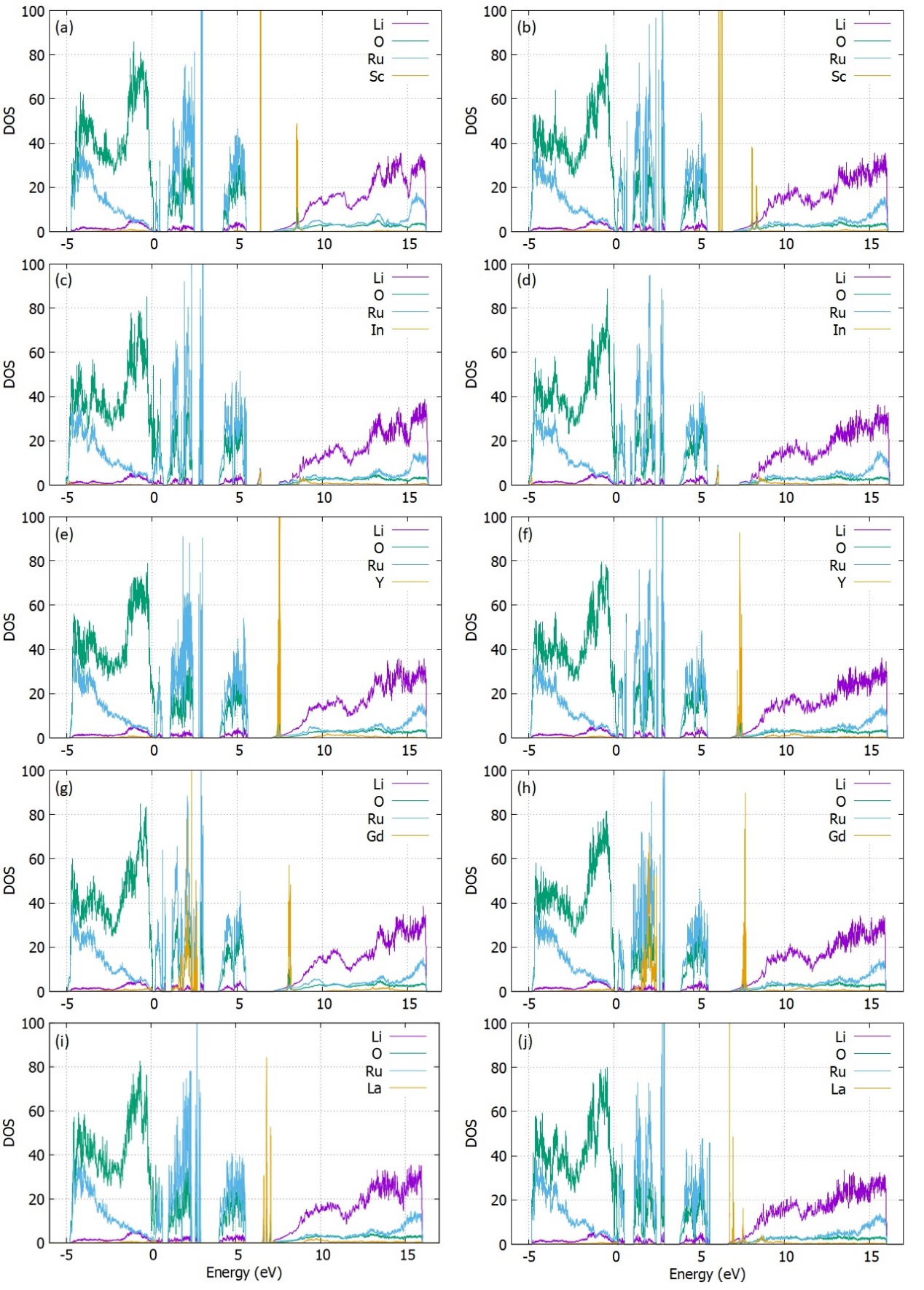


**Figure S3.** The Li_2_RuO_3_ PDOS for (a) The Sc - doped supercell (d) The Sc - doped supercell with a Li interstitial (c) The In - doped supercell (d) The In - doped supercell with a Li interstitial (e) The Y - doped supercell (f) The Y - doped supercell with a Li interstitial (g) The Gd - doped supercell (h) The Gd - doped supercell with a Li interstitial (i) The La - doped supercell (j) The La - doped supercell with a Li interstitial
